# Monolithic Low Noise and Low Zero-g Offset CMOS/MEMS Accelerometer Readout Scheme

**DOI:** 10.3390/mi9120637

**Published:** 2018-11-30

**Authors:** Yu-Sian Liu, Kuei-Ann Wen

**Affiliations:** Institute of Electronic Engineering, National Chiao Tung University, Hsinchu 300, Taiwan; stellawen@mail.nctu.edu.tw

**Keywords:** Accelerometer readout, low noise, low zero-g offset

## Abstract

A monolithic low noise and low zero-g offset CMOS/MEMS accelerometer and readout scheme in standard 0.18 μm CMOS mixed signal UMC process is presented. The low noise chopper architecture and telescopic topology is developed to achieve low noise. The experiments show noise floor is 421.70 μg/√Hz. The whole system has 470 mV/g sensitivity. The power consumption is about 1.67 mW. The zero-g trimming circuit reduces the offset from 1242.63 mg to 2.30 mg.

## 1. Introduction

Micro-electromechanical system (MEMS) products are widely used in our daily life. One of them is the MEMS accelerometer. The accelerometers have many applications in automobiles, navigation, vibration monitoring, and even portable electronics [1]. In most cases, measuring the accelerations and the additional signal processing are necessary. The CMOS (Complementary Metal-Oxide-Semiconductor)/MEMS process has the advantage of integration. The process can integrate MEMS devices as well as CMOS circuitry.

The main noise sources of readout circuit are thermal noise and flicker noise. The thermal noise comes from the random motion of electrons due to thermal effects. The 1/f noise or flicker noise is a low-frequency noise. The power spectral density (PSD) of flicker noise is inversely proportional to frequency. The accelerometer operates at low frequency. Hence, flicker noise is dominant.

Due to the process limitation, the sensing capacitor of the accelerometers at 1 g for the CMOS/MEMS process is in the order of few femto farads. The sensing signal may be damaged by electronic noise. Therefore low noise circuit is needed.

A low-noise feedforward noise reduction scheme is presented in Reference [2], which is a simple two-phase correlated double sampling (CDS) scheme to suppress the offset voltage and flicker noise. Both chopper stabilization (CS) and correlated double sampling are adopted in Reference [3]. The chopper stabilization modulate the sensing signal to high frequency. After amplification, the output chopper demodulates back to low frequency. The modulation and demodulation is simply implemented by CMOS switches driven by clock signals.

A pseudo-random chopping scheme is presented in Reference [4], which spreads the interference over a wide bandwidth, reducing its in-band portion to the level of the noise floor. Other circuit architectures such as dual-chopper amplifier (DCA), which employs two fundamental chopping clocks, have been reported in References [1,5,6,7].

Sensor readout circuits for capacitive accelerometers suffer from a signal offset due to production mismatch [8]. The offset from process variation can appear at the sensor output. It reduces dynamic range and causes the DC output level to vary from die to die [9]. The two-part correction is demonstrated in Reference [8], which consists of a capacitor array and a current digital to analog converter (DAC). A capacitor array is used to apply a signal correction by placing digitally controlled capacitors in parallel to the sensor capacitors. The second part consists of a current DAC placed within a differential amplifier to balance out asymmetric currents caused by the signal offset.

Standard CMOS process is suitable for implementing digital offset trimming. Hence, the offset trimming mechanism is presented to overcome the offset from sensor and interface circuit.

The design target refers to ADXL103. ADXL103 is a high precision, low power single-axis accelerometer with a signal conditioned voltage outputs from Analog Devices. ADXL103 measures acceleration with a full-scale range of ±1.7 g, sensitivity of 1000 mV/g, noise floor of 110 μg/√Hz, and power of 3.5 mW. ADXL103 can measure both dynamic acceleration and static acceleration. The main application is for navigation and motion detection [10].

The design target of our readout circuit is ±1 g sensing range, noise floor of 10 μg/√Hz, and power of milli-watt scale, which is suitable for navigation and motion detection. Based on our previous work [11], the UMC 0.18 μm CMOS/MEMS process is adopted for sensor and circuit implementation. This paper presents a low noise and low zero-g offset CMOS/MEMS accelerometer and readout scheme. Section 2 describes the CMOS/MEMS accelerometer and the circuit design of the low noise and low zero-g offset readout. In Section 3, describes the measurement results of the proposed readout scheme. Section 4 presents the discussion of the proposed readout scheme by comparison of performance with the state-of-the-art and presents the conclusions of this work.

## 2. Materials and Methods

### 2.1. CMOS/MEMS Accelerometer

**Figure 1:**
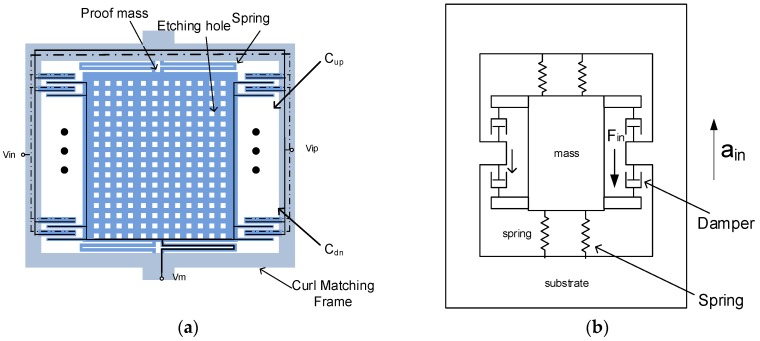

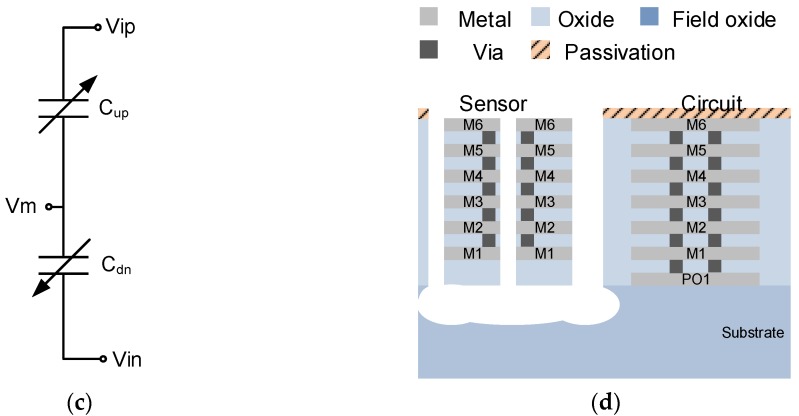
The proposed accelerometer: (**a**) The top view of the proposed accelerometer; (**b**) mechanical model of the structure; (**c**) circuit model of the structure; and (**d**) the side view of the structure.

In this work, the application-specific integrated circuit (ASIC) compatible 1P6M process of UMC 0.18 μm mixed-signal/RF CMOS process is adopted. The micromachining process is performed on the wafer of standard CMOS process. Figure 1a shows the top view of the proposed accelerometer. The proposed CMOS/MEMS accelerometer consists of proof mass, sensing fingers, single-folded springs, and a curl-matching frame. It is equivalent to a second-order mass-spring-damper mechanical model, as in Figure 1b. The displacement is transformed into capacitance ΔC by sensing fingers. The circuit model in Figure 1c is simulated with readout circuit. The circuit is simulated in Cadence design environment by Spectre simulator. The side view of the CMOS process with micromachining post process is shown in Figure 1d.

### 2.2. Readout Circuit Design

The readout circuit has two main parts. The first part is a low noise unit and contains the main amplifier and pre-amplifier. The low noise chopper architecture and telescopic topology is developed to achieve low noise. The second part is a sensor-trimming unit that is an 8-bit trimming capacitor.

**Figure 2:**
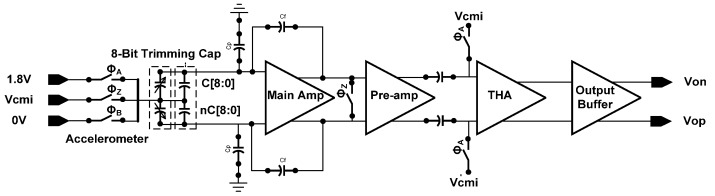
The system architecture.

The architecture is shown in Figure 2. The sensing signal is modulated to 333 kHz and passes through amplification stages, track-and-hold amplifier (THA), output stage, and band limiting RC filter. The overall performance summery is listed in Table 1.

**Figure 3:**
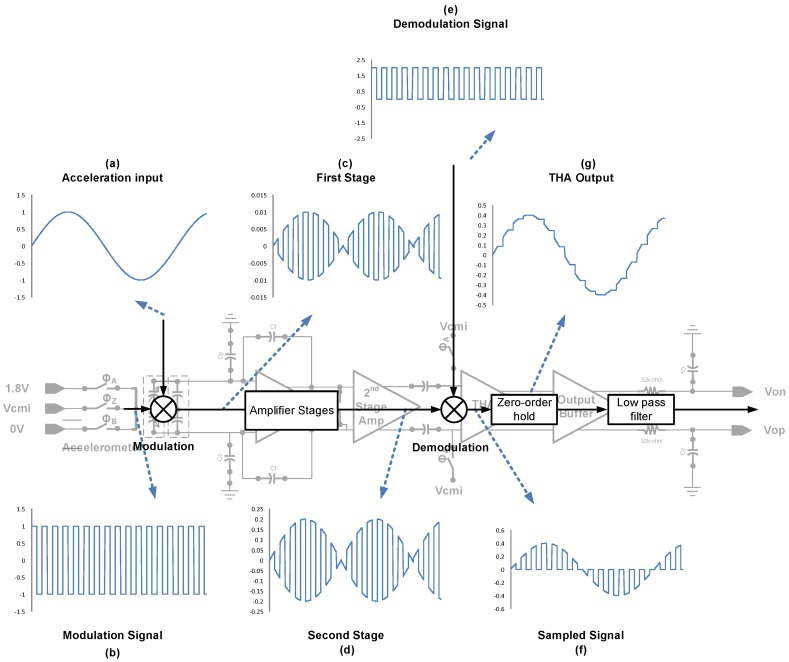
System function blocks with simplified modulation signal of the proposed readout circuit.

Figure 3 shows the working principle of the sensor readout with the simplified modulation signal, which can be found in Figure 3b. The modulation frequency is 333.33 kHz. The sensing signal in Figure 3a is modulated by modulation clock signal and passes through amplifier stages in Figure 3c,d. The demodulation is achieved by track-and-hold stage, which is equivalent to multiply the demodulation signal in Figure 3e. The demodulated signal in Figure 3f passes through the zero-order hold and the signal in Figure 3g is obtained.

#### 2.2.1. Low Noise Chopper Architecture

Since flicker noise is inversely proportional to frequency, the operation frequency determines the noise performance. The chopper architecture modulates the signal to chopping frequency to suppress flicker noise. The quantitative analysis is carried out at the transistor level to verify the effectiveness of the proposed architecture.

**Figure 4:**
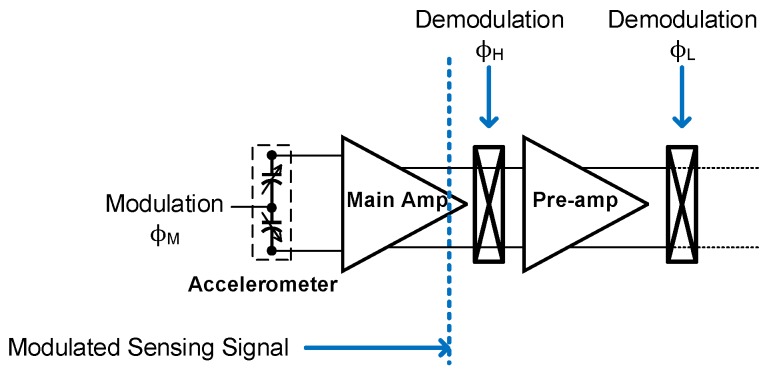
The simplified architecture in Reference [1].

The sensing signal is modulated to 333.33 kHz by the switches (ϕ_A_, ϕ_Z_ and ϕ_B_), which is known as signal chopping. In this work, both the main amplifier and pre-amplifier are working at high frequency (at 333.33 kHz chopping frequency). For the conventional design in Reference [1], the sensing signal is demodulated using ϕ_H_ (at 1 MHz chopping frequency) in Figure 4. After demodulation by ϕ_H_, the signal is further boosted by an amplifier.

**Figure 5:**
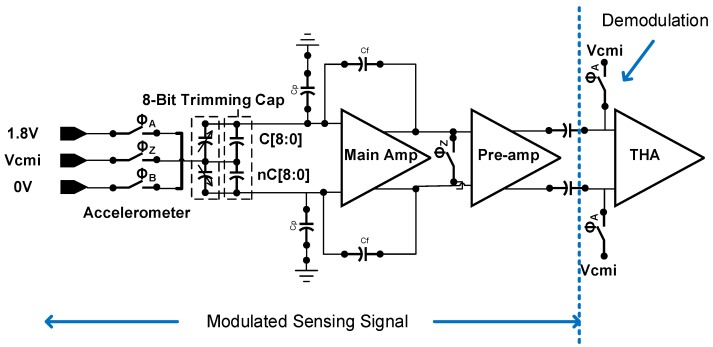
The proposed architecture for noise reduction.

The proposed low noise interface circuit is presented in Figure 5. The modulated sensing signal is first amplified by main amplifier and further boosted by the pre-amplifier. The amplified signal is demodulated by ϕ_A_.

The noise figure *F* of the network is defined as the ratio of the available signal-to-noise ratio at the signal-generator terminals to the available signal-to-noise ratio at its output terminals as the following equation [12].

(1) $$F=\frac{SNR_{in}}{SNR_{out}}$$

Total noise figure of the whole system can be expressed by Friis’ Formula:

(2) $$F_{sys}=F_{1}+\frac{F_{2}-1}{G_{1}}+\frac{F_{3}-1}{G_{1}G_{2}}+\frac{F_{4}-1}{G_{1}G_{2}G_{3}}+\ldots +\frac{F_{n}-1}{G_{1}G_{2}G_{3}\ldots G_{n-1}}$$

where *F_n_* is the noise figure for the *n*-th device and *G_n_* is the power gain (linear, not in dB) of the *n*-th device. The design target is lower than the whole system *F_sys_*. For the two amplifier stages, the noise of main amplifier is *F*_1_, the power gain of main amplifier is *G*_1_, and the noise of pre-amplifier is *F*_2_. The noise figure of the third stage *F*_3_ will be divided by the gain of the first two stages (the *G*_1_*G*_2_ term). Thus, the noise figures of the first two stages must be considered [12]. The simplified noise figure is given by:

(3) $$F_{sys}\approx F_{1}+\frac{F_{2}-1}{G_{1}}$$

Two strategies are applied to lower the noise figure *F_sys_*. The proposed circuit architecture minimize the *F*_1_ and *F*_2_ terms. First, modified the circuit architecture operates the second stage amplifier at 333.33 kHz to lower the *F*_2_ term, which reduces noise contribution from the second stage amplifier. Second, the noise factor of the first amplifier *F*_1_ is significant for the readout circuit since the *F*_1_ term is directly added to *F_sys_*. The gain *G*_1_ is determined by the overall sensitivity. *G*_1_ is around 7.88 V/V.

**Figure 6:**
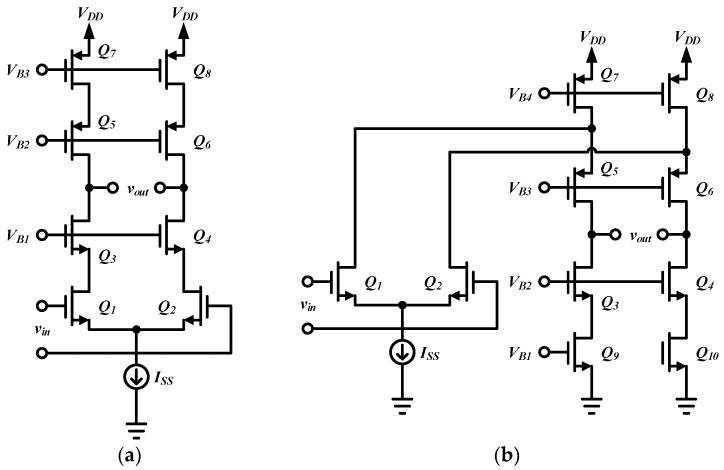
Schematic of telescopic amplifier.

The telescopic amplifier is shown in Figure 6a, *Q*_1_ and *Q*_2_ form the input differential pair, and *Q*_3_–*Q*_6_ are the cascode transistors. Cascading transistors increase the voltage gain at the cost of output voltage headroom. Since the output swing requirements are very small at the first stage, on the order of several millivolts, a telescope may be used. For telescopic topology, the *Q*_1_, *Q*_2_, *Q*_7_, and *Q*_8_ are the primary noise sources. The folded-cascode topology is a popular amplifier architecture as in Figure 6b. *Q*_1_ and *Q*_2_ form the input differential pair, and *Q*_5_ and *Q*_6_ are the cascode transistors, which are folded, as compared to telescopic topology. For folded-cascode topology, the *Q*_1_, *Q*_2_, *Q*_7_, *Q*_8_, *Q*_9_, and *Q*_10_ are the primary noise sources. Assuming the transistors exhibit similar noise levels, folded-cascode topology suffers from greater noise than its telescopic counterpart. The telescopic topology is desirable since it has fewer noise-contributing transistors, and hence F_1_ is reduced.

Spectre PNoise simulation is used for noise characterization. The PNoise simulation gives the noise response of main amplifier and pre-amplifier. Table 2 shows the comparison of noise and power at each stage. For main amplifier, Reference [1] modulates the sensing signal to 1 MHz, while the proposed architecture modulates to 333.33 kHz. For pre-amplifier, Reference [1] demodulates the sensing signal to 20 kHz, while the proposed architecture is still working at 333.33 kHz. Comparing the two frequency arrangements, the proposed architecture has 8% less noise than Reference [1] at the cost of 10% more power consumption. The simulation results verify the effectiveness of the proposed reduction architecture.

#### 2.2.2. Low Zero-g Offset Design

**Figure 7:**
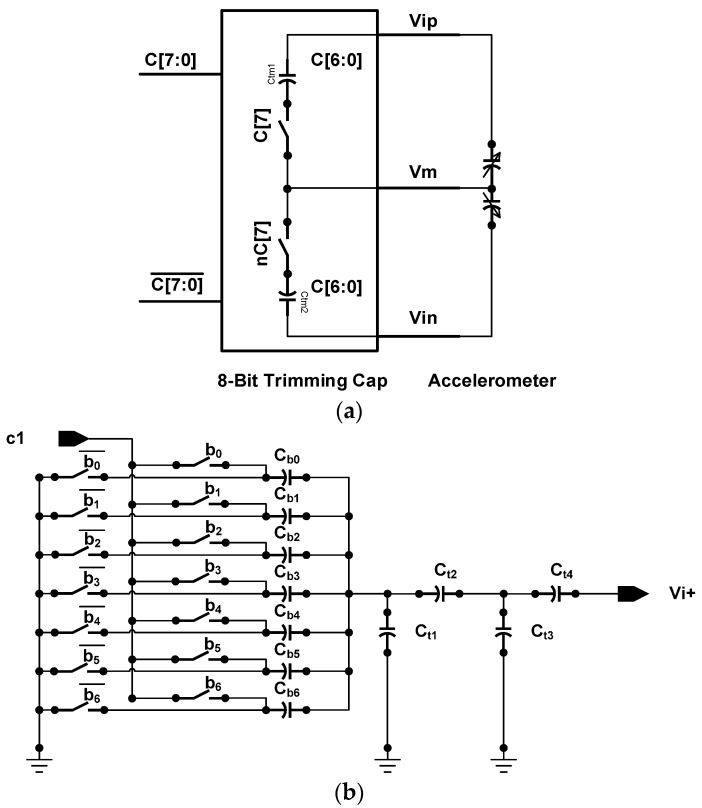
Schematic of 8-bit trimming capacitance: (**a**) The 8-bit trimming capacitor with the CMOS/MEMS accelerometer circuit model; and (**b**) the 7-bit segmented split capacitor structure.

The sensing capacitive mismatch needs to be compensated. Small capacitor in sub femto farad scale is placed in parallel with the sensing capacitors to cancel the sensor offsets. A segmented split capacitor structure is proposed to realize small capacitor, as in Figure 7. Figure 7b shows the 7-bit trimming capacitance. The most significant bit (MSB) C[7] controls the switch in Figure 7a, which determines adding trimming capacitance to the upper plane or lower plane of the accelerometer. The *C_tm_*_1_ and *C_tm_*_2_ are the 7-bit trimming capacitance in Figure 7b. Trimming capacitance is estimated by using the equation below:

(4) $$C\approx \frac{b_{0}C_{b0}+b_{1}C_{b1}+b_{2}C_{b2}+b_{3}C_{b3}+b_{4}C_{b4}+b_{5}C_{b5}+b_{6}C_{b6}}{(C_{b0}+C_{b1}+C_{b2}+C_{b3}+C_{b4}+C_{b6})+C_{t1}}(\frac{C_{t2}}{C_{t2}+C_{t3}})C_{t4}$$

The ratio of capacitance *C_t_*_2_ and *C_t_*_3_ make the overall capacitance *C* smaller to get sub femto farad scale capacitance.

## 3. Results

**Figure 8:**
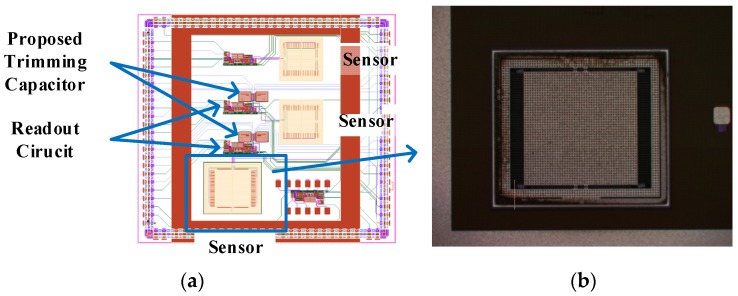
The chip layout and die photo.

The circuit is implemented in UMC 0.18 μm process. In this work, low noise readout scheme is presented. The trimming capacitor is added for zero-g offset compensation. The die photo and chip layout is shown in Figure 8.

### 3.1. CMOS/MEMS Accelerometer

**Figure 9:**
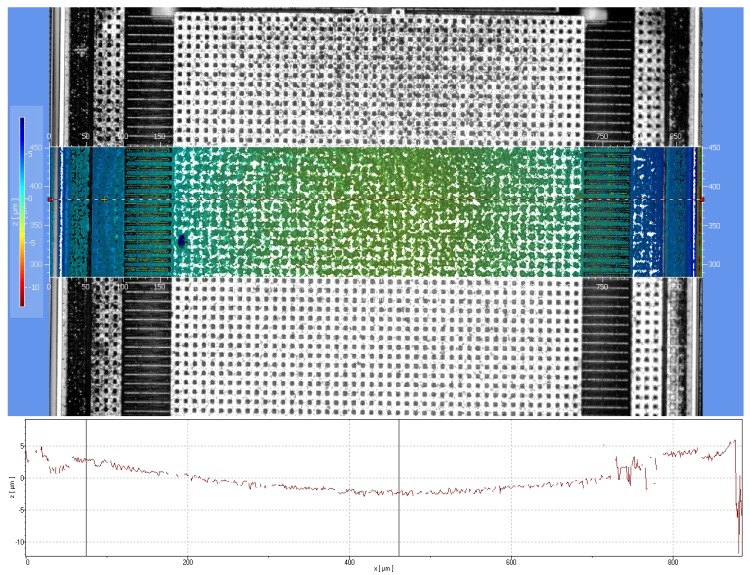
Cross section of the accelerometer.

The dry-etch-based post-process are used after standard CMOS process for microstructure fabrication. Figure 9 shows the cross section of the CMOS/MEMS accelerometer. The curl matching frame and the proof mass of accelerometer have the same curling.

### 3.2. Low Noise Design

**Figure 10:**
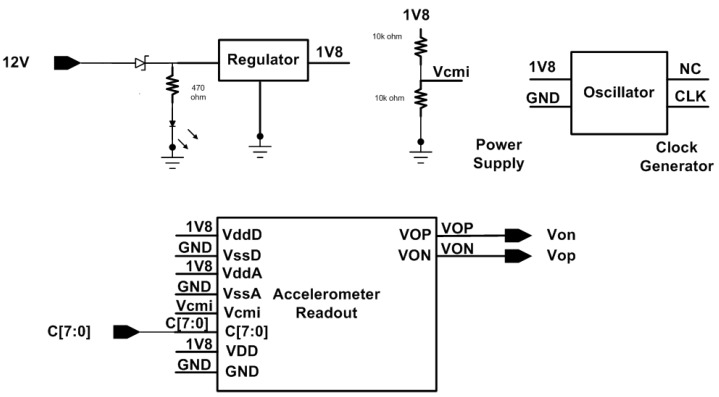
Simplified evaluation board schematics for accelerometer and readout circuit characterization.

**Figure 11:**
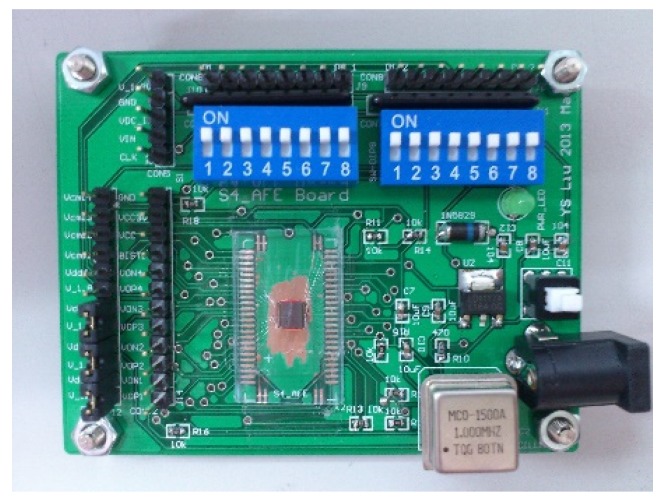
Evaluation board photo.

Figure 10 shows the evaluation board schematic for acceleration readout measurement. The fabricated chip directly mounts on the printed circuit boards. On board oscillator generates 1 MHz clock for acceleration readout. The 1.8 V supply is generated by regulator for digital power (V_ddD_) and analog power (V_ddA_). The calibration readout is controlled by on-board switches. The fabricated chip directly mounts on the printed circuit boards, as shown in Figure 11.

#### Noise Considerations in Board Design

A digital circuit can produce noise at 1 MHz. Circuit noise decoupling capacitors are added at power line for digital noise reduction (1 MHz) (power line filter). Since the power line is 60 Hz, which is near 100 Hz of the sensing signal and cannot be easily filter by conventional filter. Power line noise is isolated by using battery power. The battery power passes though voltage regulator into readout circuit. The voltage regulator LM1117 is adopted, which reported RMS output noise is 0.003% of V_OUT_ at frequency 10 Hz ≤ f ≤ 10 kHz, where V_OUT_ is 1.8 V.

**Figure 12:**
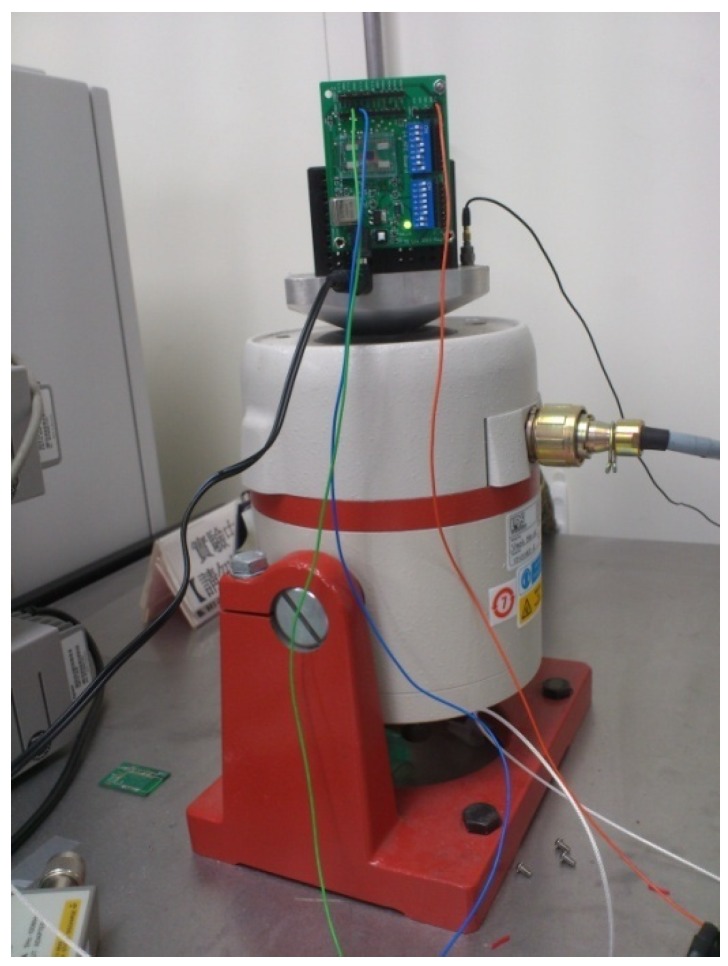
Measurement setup for accelerometer and readout circuit characterization.

**Figure 13:**
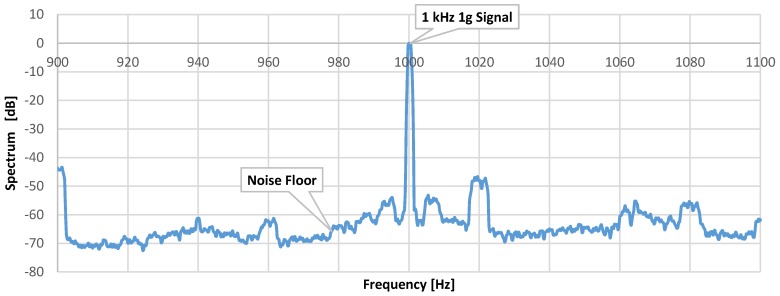
The output noise spectrum.

The evaluation board is placed on the LDS V408 shaker, as shown in Figure 12 for noise and sensitivity measurement. The shaker generates 1 g signal 1 kHz acceleration input. Figure 13 shows the spectrum of output voltage at the excitation. The noise floor is 421.70 μg/√Hz. The signal-to-noise ratio (SNR) is around 67.5 dB.

**Figure 14:**
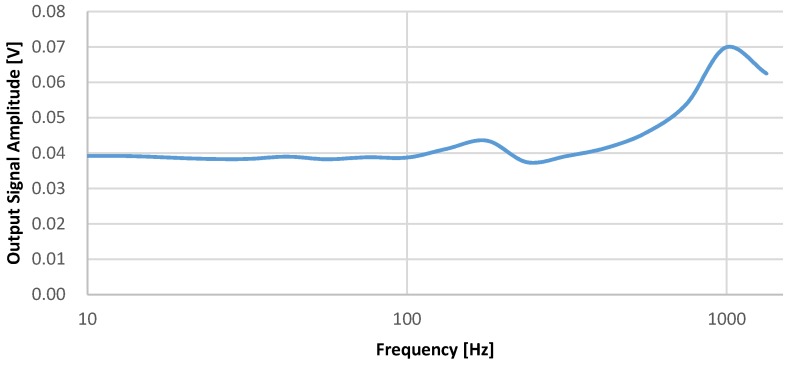
The frequency response of the system.

The sensitivity of the system is characterized for the two aspects, linearity and frequency response. The shaker generates 0.25 g signal to characterize the frequency response of the system as in Figure 14. The frequency range from 10 Hz to 1333.33 Hz is limited by the shaker. For the frequency around 1 kHz, the sensitivity increases due to the resonance of accelerometer.

**Figure 15:**
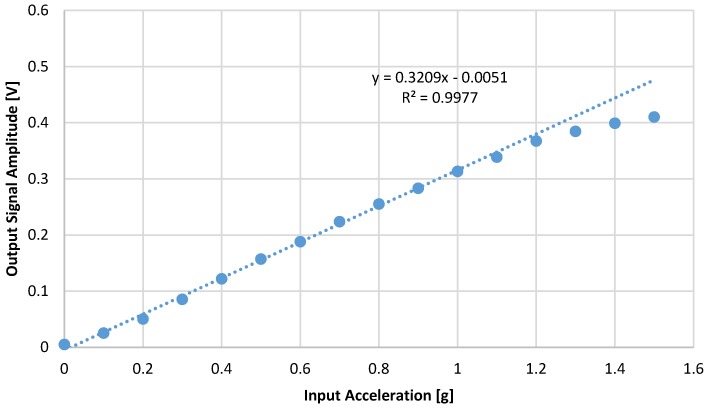
The output signal amplitude versus acceleration.

The sensing range of readout circuit is designed for ±1 g. The readout circuit is characterized using 1 kHz signal from zero to 1.5 g as in Figure 15. The linear regression is performed for zero to 1 g input signal. For a signal larger than 1 g, the output saturates and deviates from linear operation.

### 3.3. Low Zero-g Offset Design

The trimming capacitor is controlled by the digital value from the evaluation board to eliminate the zero g offset. The zero-g offset of the system is characterized for the two aspects, static and dynamic operation.

**Figure 16:**
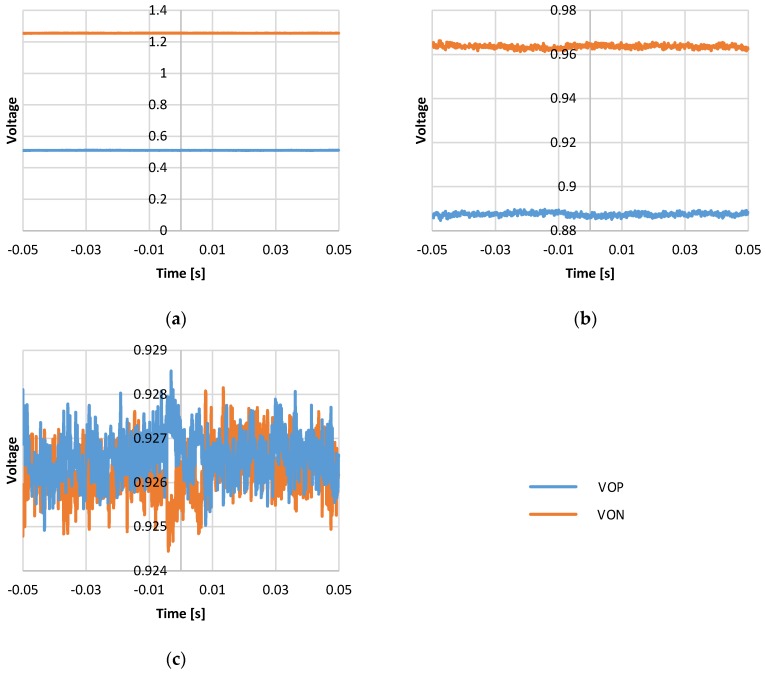
Output measurement without external excitation for various offset trimming configuration: (**a**) Trimming capacitor with 8’b0000_0000 configuration; (**b**) trimming capacitor with 8’b1111_1111 configuration; and (**c**) trimming capacitor with 8’b1001_0000 configuration.

For static operation, the system output measured without external excitation that is the zero g output. For the ideal case, the zero-g output should be zero. The difference of positive output (VOP) and negative output (VON) represents the accelerometer readout. The differential output of the sensing signal VOP and VON should be the same. Figure 16 shows the output voltage with different configurations of the trimming capacitor. For the 8’b0000_0000 configuration, the 0 fF trimming capacitor is in parallel to the sensor capacitors, which stands for zero g offset value without trimming. The circuit output is saturated. The zero-g offset is 745.06 mV, as in Figure 16a. For the 8’b1111_1111 configuration, the maximum trimming capacitance is in parallel to the sensor capacitors. The offset is 77.08 mV, as in Figure 16b. For the 8’b1001_0000 configuration, the trimming capacitance is in parallel to eliminate the zero g offset. The offset is reduced from 745.06 mV to 1.38 mV. That is, the zero g offset is reduced from 1242.63 mg to 2.30 mg, as in Figure 16c.

**Figure 17:**
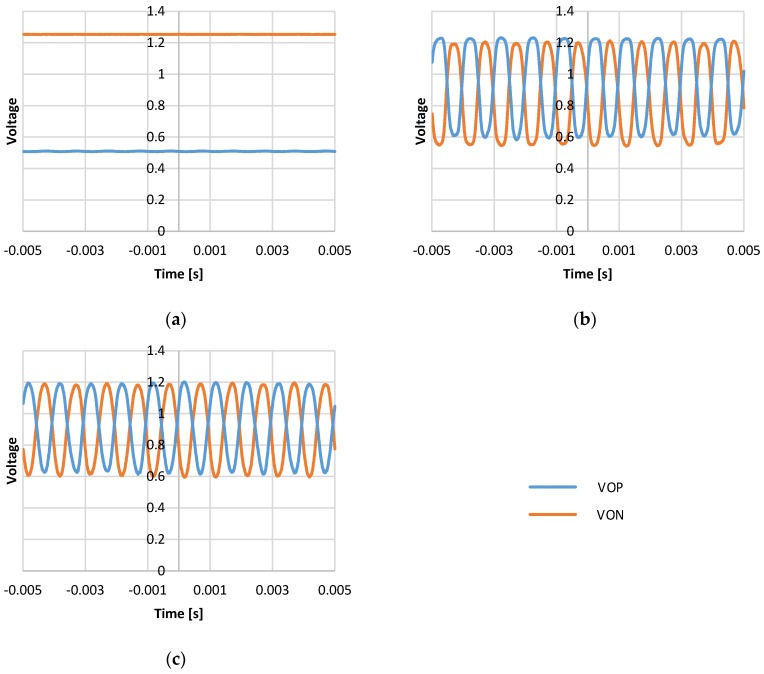
Output measurement with 1 g 1 kHz excitation for various offset trimming configuration: (**a**) Trimming capacitor with 8’b0000_0000 configuration; (**b**) trimming capacitor with 8’b1111_1111 configuration; and (**c**) trimming capacitor with 8’b1001_0000 configuration.

For dynamic operation, the excitation of 1 g 1 kHz is applied with different configurations of the trimming capacitor. For the 8’b0000_0000 configuration, the circuit output is saturated. Sensitivity is degraded to 1.61 mV/g, as in Figure 17a. For the 8’b1111_1111 configuration, the sensitivity is around 706.32 mV/g. The output exhibits nonlinear distortion, which is obviously undesirable, as in Figure 17b. For the 8’b1001_0000 configuration, the trimming capacitance is in parallel to eliminate the zero g offset. The measurement shows sensitivity around 599.58 mV/g, as in Figure 17c.

## 4. Discussion and Conclusions

A monolithic low noise and low zero-g offset CMOS/MEMS accelerometer and readout scheme in standard 0.18 μm CMOS mixed signal UMC process is presented. For 1 g 100 Hz acceleration input, the whole system has 470 mV/g sensitivity. The power consumption is about 1.67 mW. Table 3 compares the performance of the work proposed here to the state-of-the-art. Comparing with Reference [2], using the same 0.18 μm process node, the noise floor and zero g offset is reduced, while the overall power consumption is increased.

The low noise chopper architecture and telescopic topology is developed to achieve low noise. The experiments show that noise floor is 421.70 μg/√Hz. The trimming capacitors are used for offset calibration. The zero g trimming circuit reduces the offset from 1242.63 mg to 2.30 mg.

## Figures and Tables

**Table 1:** System specifications.

Specifications	Post-Sim	Measurement
System frequency (MHz)	1	1
Chopper frequency (kHz)	333.33	333.33
Overall sensitivity (mV/g) (at 1 g 100 Hz)	434.93	470
Noise (μg/√Hz) (at 1 g 100 Hz)	10	421.70
Resonance Frequency (kHz)	4.16	1.25
Brownian noise (μg/√Hz)	7.9	-
Sensing Range (g)	±1	±1
Power (mW)	2	1.67

**Table 2:** Analysis and comparison of noise and power at the two stages.

Stage	This Work			Reference [1]		
	Signal Frequency (Hz)	Power (μW)	Noise (μg/√Hz)	Signal Frequency (Hz)	Power (μW)	Noise (μg/√Hz)
Main Amp.	333.33 kHz	37.18	40.45	1 MHz	29.93	30.18
Pre-Amp	333.33 kHz	40.17	59.84	20 kHz	39.40	171.80
Total		77.34	48.04		69.33	51.97

**Table 3:** Comparison of the proposed readout scheme to the state-of-the-art.

Parameters	[3]	[4]	[13]	[2]	This Work
Sensing Range (g)	±4	±49	±30	±8	±1
Noise Floor (μg/√Hz)	930	380	1	970	421.70
Zero-g Offset (mg)	N/A	N/A	N/A	±33	2.30
Supply Power (V)	1.2	1.9/3.3 ^1^	1.5	1	1.8
Power (W)	25.44 μ	1.4 m	2.7 m	181 n	1.67 m
Chip Area (Readout Circuit) (mm^2^)	1.73	1.1	10.9	1.14	1.23
Process	0.25 μm CMOS process	0.18 μm CMOS process	0.35 μm CMOS process	0.18 μm CMOS process	UMC 0.18 μm CMOS/MEMS

^1^ A 1.9-V supply is regulated from an external 3.3-V supply.
